# Unsupervised clustering identifies distinct phenotypes in acute myocardial infarction: insights from the FAST-MI 2015 registry

**DOI:** 10.3389/frai.2026.1886402

**Published:** 2026-08-19

**Authors:** Vincent Bataille, Loïc Panh, Etienne Puymirat, Guillaume Cayla, Pascal Motreff, Gilles Lemesle, François Schiele, Tabassome Simon, Nicolas Danchin, Jean Ferrières

**Affiliations:** 1 INSERM UMR 1295, CERPOP, Toulouse Rangueil University Hospital, Toulouse University School of Medicine, Toulouse, France; 2 ADIMEP (Association pour la Diffusion de la Médecine de Prévention), Toulouse, France; 3 Department of Cardiology, Clinique Pasteur, Toulouse, France; 4 Department of Cardiology, Assistance Publique-Hôpitaux de Paris (AP-HP), Hôpital Européen Georges Pompidou, Paris, France; 5 Department of Cardiology, University Hospital of Nîmes, University of Montpellier, Nîmes, France; 6 Department of Cardiology, University Hospital of Clermont-Ferrand, Clermont-Ferrand, France; 7 Department of Cardiology, Heart and Lung Institute, University Hospital of Lille, Lille, France; 8 French Alliance for Cardiovascular Trials (FACT), Paris, France; 9 Department of Cardiology, University Hospital of Besançon, University of Franche Comte, SINERGIES, Besançon, France; 10 Department of Clinical Pharmacology, Hôpital Saint-Antoine, Assistance Publique-Hôpitaux de Paris (AP-HP), Paris, France; 11 Department of Cardiology, Hôpital Saint Joseph, Paris, France; 12 Department of Cardiology, Toulouse Rangueil University Hospital, Toulouse, France

**Keywords:** acute coronary syndrome, cluster analysis, myocardial infarction, precision medicine, risk stratification

## Abstract

**Background:**

Current classification of Acute Myocardial Infarction (AMI) into ST-elevation (STEMI) and non-ST-elevation (NSTEMI) myocardial infarction does not fully reflect the clinical heterogeneity of patients.

**Objectives:**

To identify clinically meaningful phenotypes of AMI patients using an unsupervised clustering approach and assess their associations with management strategies and long-term outcomes.

**Methods:**

Hierarchical agglomerative clustering using Ward’s method was performed in 4,947 patients from the French FAST-MI 2015 nationwide registry. Clustering was based on baseline clinical, demographic, and laboratory features. Between-cluster differences in baseline characteristics, management, in-hospital complications, and 1-year mortality were analyzed.

**Results:**

Four phenotypic clusters were identified. Cluster 1 (n = 1,488) gathered older patients, predominantly men, with typical coronary risk factors, intermediate risk profile, and balanced STEMI/NSTEMI presentations. Cluster 2 (*n* = 1,305) gathered older polymorbid patients, 45% were women, 60% with NSTEMI presentation, with limited use of invasive strategies (71%), and a high 1-year mortality. Cluster 3 (n = 815) gathered young (mean age 44 years), predominantly male patients, with a high prevalence of smoking (73%) and few comorbidities, 62% STEMI, high rates of revascularization, and excellent prognosis. Cluster 4 (*n* = 1,339) was close to Cluster 3 with a more metabolic profile with higher rates of obesity, diabetes, and dyslipidemia, also with favorable outcomes. In-hospital complications were more frequent in Clusters 1 and 2. One-year mortality was lowest in Clusters 3 and 4 (1.5 and 2.3%), intermediate in Cluster 1 (6.0%), and highest in Cluster 2 (15.7%).

**Conclusion:**

Unsupervised clustering identified four clinically relevant AMI phenotypes with distinct characteristics, management patterns, and prognoses. This data-driven classification highlights the diversity of AMI presentations and may offer complementary insights for patient profiling and risk stratification.

## Introduction

The management of AMI is currently guided by a pathophysiological approach differentiating non occlusive and occlusive artery thrombosis inducing different 12-lead electrocardiogram changes. This dichotomy, notably differentiating STEMI from NSTEMI is the basis of major clinical guidelines and treatment algorithms. International boards recommend immediate coronary reperfusion in patients with STEMI due to occlusive thrombosis, whereas NSTEMI patients management depends on clinical symptoms and risk score assessment (Byrne et al., 2023). While this framework has enabled rapid decision-making and improved access to reperfusion therapy in STEMI patients, it may not fully capture the clinical complexity and heterogeneity of the AMI population.

Thanks to emergency coronary reperfusion strategies and optimized medical therapies, contemporary cardiology in industrialized countries has considerably improved outcomes and prognosis of coronary heart disease (CAD) (Puymirat et al., 2017). However, we currently observe a lack of efficiency in advances in both medical strategies (Sabatine et al., 2017) and the development of new technical approaches for the management of CAD (Mezilis et al., 2010; Van Veelen et al., 2021). Furthermore, standard statistical models may struggle to account for the wide range of patient’s characteristics that influence prognosis and treatment response. As a result, treatment recommendations often rely on simplified classifications that may not reflect the complexity of underlying disease profiles. For example, STEMI in an elderly diabetic woman with multivessel calcified coronary disease and heart failure constitutes a very different clinical scenario from a young hypercholesterolemic man presenting with STEMI due to a single-vessel occlusion. Yet, both are typically managed within the same binary classification system. Conventional analyses are sometimes unable to integrate multidimensional data in a way that reveals novel phenotypic patterns or predicts outcomes more accurately.

Unsupervised clustering offers a promising alternative as these methods are likely to reveal natural groupings within complex datasets, free from some biases inherent in predefined categories or clinician-driven classifications. Clustering has been widely applied in fields such as oncology and critical care to identify data-driven phenotypes (Loftus et al., 2022; Jacquemyn et al., 2024), but its application in CAD remains limited (Banerjee et al., 2021).

The aim of this study was to identify clinically meaningful phenotypes using an unsupervised clustering approach applied to the large French national FAST-MI 2015 cohort of patients with AMI, and to explore their association with early management strategies and long-term outcomes.

## Methods

### Study population

Subjects were 5,291 patients admitted in a Cardiology Intensive Care Unit (CICU) following an AMI and were included in the French Registry of Acute ST-elevation or Non-ST-elevation Myocardial Infarction 2015 cohort (FAST-MI 2015). The FAST-MI 2015 (Bouisset et al., 2021) registry is a nationwide, prospective, multicenter study conducted across 204 hospitals or private clinics with a CICU (NCT02566200). Patients included were consecutive adults admitted for AMI within 48 h of symptom onset, with confirmation by elevated cardiac biomarkers and supporting clinical or electrocardiographic findings. The registry was designed to be representative of routine clinical practice, including university hospitals, community hospitals, and private clinics. All centers were encouraged to enroll patients consecutively. Patients were enrolled between October and December 2015.

### Data collection

Data were recorded from the source patient files on an electronic case record form by dedicated research technicians who went to each center on a weekly basis. In the case of incomplete data from the source files, the research technicians contacted the local investigator to obtain missing information. Detailed data regarding patient demographics, cardiovascular history and cardiovascular risk factors, clinical presentation, pre-hospital and in-hospital therapeutic management, medical treatments (pre-existing, during in-hospital stay and at discharge), in-hospital complications, and on discharge were collected using standardized electronic case report forms.

### Follow-up

Patient follow-up was performed at 6 months, 1 year, and then on a yearly basis by dedicated research technicians looking for vital status and any occurrence of a morbid event leading to hospitalization, following a sequential procedure: request from town halls of birth and/or national civil status services, then contact with the patient’s general practitioner and/or cardiologist, then contact with the patient or his/her relatives. All recorded events leading to any hospitalization were reviewed and classified by a 3-person critical events committee.

### Statistical analysis

Clustering was performed using hierarchical agglomerative clustering with Ward’s linkage method. This unsupervised clustering approach begins by considering each individual as a separate cluster and iteratively merges the two clusters that minimize the increase in within-cluster variance (Ward’s minimum variance criterion). The method favors the formation of compact and homogeneous clusters. Only baseline variables available at admission or reflecting pre-existing patient characteristics were included in the clustering procedure; variables related to in-hospital management, therapeutic decisions, or outcomes were intentionally excluded from the clustering process and analyzed descriptively thereafter. Then, baseline variables used for clustering included the type of healthcare facility (public hospital, private clinic…), gender, age, cardiovascular risk factors, history of cardiac disease, comorbidities, ongoing treatments at the time of the qualifying AMI, type of AMI (STEMI/NSTEMI), and admission laboratory values (CRP and creatinine). Missing values for clustering variables CRP and creatinine were retained through an “unknown” category. This approach was chosen because missingness was considered likely to reflect clinical decision-making and patient characteristics rather than being completely random. Clustering was performed directly on the selected variables without prior dimensionality reduction. The number of clusters retained was primarily guided by dendrogram inspection and supported by silhouette analysis across a range of cluster solutions (k = 2–6). Variable contributions to cluster assignment were assessed using Cramer’s V. To assess the robustness of the clustering solution, a sensitivity analysis using k-means clustering was performed.

Categorical variables were presented as numbers and percentages, continuous variables as means ± standard deviations, and as median + interquartile range when skewed. Descriptive statistics were reported based on available data. When the proportion of missing values for a variable was not negligible and notably for laboratory values, a dedicated “unknown” category was created.

Between-cluster comparisons were performed using ANOVA or Kruskal–Wallis tests for continuous variables, and chi-square tests for categorical variables. Kaplan–Meier survival curves were generated to assess time-to-event outcomes, and log-rank tests were used for unadjusted comparisons. Losses to follow-up were censored at the date of last contact. Within clusters, factors associated with one-year mortality were investigated using a two-step approach. First, univariable analyses were performed to identify variables associated with one-year mortality. Variables with a *p*-value <0.15 in univariable analyses were then entered into multivariable Cox proportional hazards models using a backward stepwise selection procedure to identify independent predictors.

All analyses were two-sided, and *p*-values < 0.05 were considered statistically significant. All analyses were performed using Stata (StataCorp. 2023. *Stata Statistical Software: Release 18.* College Station, TX: StataCorp LLC.).

## Results

### Clusters

**Figure 1:**
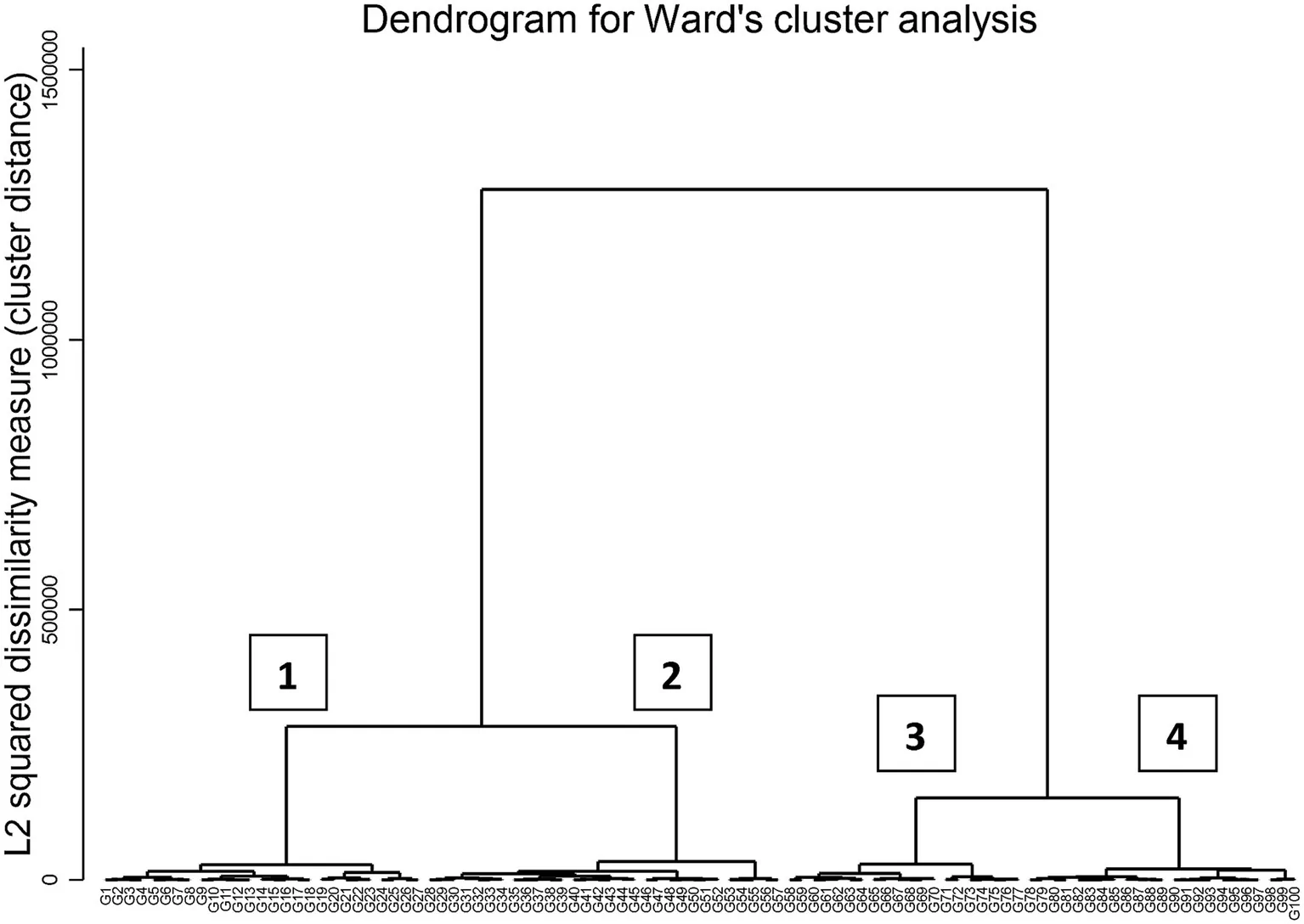
Dendrogram from agglomerative hierarchical clustering using Ward’s method in the FAST-MI 2015 cohort (*n* = 4,947; last 100 clusters are displayed).

Of the 5,291 patients enrolled in FAST-MI 2015, 4,947 (93.5%) were included in the clustering analysis and 344 (6.5%) were excluded because of missing data (Supplementary Figure 1). The proportion of missing values for all candidate variables used in the study is reported in Supplementary Table 1; and main characteristics of included and excluded patients are presented in Supplementary Table 2. Excluded patients were older, more frequently female, more often presented with STEMI and Killip class III–IV, and had lower revascularization rates and higher one-year mortality. Figure 1 shows the dendrogram obtained through hierarchical agglomerative clustering, illustrating the successive steps of merging observations into clusters. Based on the structure of this dendrogram, four clusters were retained as they represented the most distinct grouping before major linkage fusion occurred. Average silhouette coefficients were 0.490, 0.392, 0.360, 0.264 and 0.239 for k = 2, k = 3, k = 4, k = 5 and k = 6, respectively. A four-cluster solution was retained based on dendrogram inspection, interpretability, and clinical relevance. Age was the strongest discriminating variable (Cramer’s V = 0.76), followed by smoking status (V = 0.52), hypertension (V = 0.33), inaugural MI (V = 0.29), prior diuretic use (V = 0.26), gender (V = 0.25), and comorbidity burden (V = 0.22) (Supplementary Figure 2). To assess robustness, a sensitivity analysis using k-means clustering was performed and yielded a comparable four-cluster solution, with a high degree of concordance in patient assignment across these two methods (93, 99, 88 and 94%, respectively, for clusters 1, 2, 3 and 4). Moreover, another sensitivity analysis restricted to patients with complete CRP and creatinine data (*n* = 3,780) yielded a four-cluster solution with broadly similar clinical and prognostic characteristics (Supplementary Table 3). Likewise, although STEMI/NSTEMI status was included among the clustering variables, a sensitivity analysis excluding this variable yielded an overall concordance of 86% with the primary four-cluster solution. Concordance was 84, 99, 100 and 67% for Clusters 1, 2, 3 and 4, respectively. The elderly polymorbid (Cluster 2) and the young smoking (Cluster 3) groups remained highly stable, and most of the discordance was related to a partial redistribution of some patients from Cluster 4 to Cluster 3, while preserving the overall four-cluster structure.

### Baseline characteristics

The baseline characteristics of these 4 balanced phenotype groups are summarized in Table 1. Cluster 1 (*n* = 1,488) gathered patients with a typical CAD profile, of moderate severity: mean age was 68 years ± 4, 76.7% were men, with balanced risk factor profile, 46.8% had a STEMI presentation. Compared to this first group, Cluster 2 (*n* = 1,305) was marked by the over-representation of women (44.8%), increased age (mean age 82 years ± 5), and comorbidities. Most of the patients in this group had a NSTEMI presentation (60.8%). Patients from Clusters 3 (*n* = 815) and 4 (*n* = 1,339) shared several common characteristics and were younger (mean age 44 ± 5 and 57 ± 3 years old respectively), with few extra cardiac life-threatening comorbidities (16.7 and 19.3%), inaugural event for 83.7 and 73.8%, predominant STEMI presentation (61.8 and 55.6%) and a higher initial Left Ventricular Ejection Fraction (LVEF) compared to Clusters 1 and 2 (53% vs. 50 and 49%, respectively). However, compared with Cluster 3, patients from Cluster 4 displayed a more pronounced metabolic profile, notably with a higher prevalence of diabetes (8.6% in Cluster 3 and 18.3% in Cluster 4) and dyslipidemia (32.6 and 43.2%). Most of patients in these two groups were men (81.1 and 81.8%), with notably high frequencies of active smoking (73.0 and 57.1%), and obesity (28.4 and 23.6%).

**Table 1:** Baseline demographic, clinical and biological characteristics of patients across the four identified clusters.

	Cluster 1 (*n* = 1,488)		Cluster 2 (*n* = 1,305)		Cluster 3 (*n* = 815)		Cluster 4 (*n* = 1,339)		*p*	Overall (*n* = 4,947)	
	*n*	%	*n*	%	*n*	%	*n*	%		*n*	%
Type of establishment *									0.536		
University hospital	583	39.2	502	38.5	320	39.3	545	40.7		1,950	39.4
General hospital	687	46.2	622	47.7	397	48.7	621	46.4		2,327	47.0
Private clinic	218	14.7	181	13.9	98	12.0	173	12.9		670	13.5
Socio-demographics											
Female gender *	346	23.3	584	44.8	153	18.9	243	18.2	< 0.001	1,326	26.8
Age (years) *	68 **	+/− 4	82	+/− 5	44	+/− 5	57	+/− 3	< 0.001	65	+/− 14
*n*	1,488		1,305		815		1,339			4,947	
Cardiovascular risk factors											
BMI (kg/m^2^) *									<0.001		
< 25	496	34.9	558	45.1	249	31.3	428	32.9		1,731	36.4
25–29	583	41.1	476	38.5	320	40.3	565	43.5		1,944	40.9
≥ 30	341	24.0	202	16.3	226	28.4	306	23.6		1,075	22.6
Weight (kg)	79	+/− 16	71	+/− 14	83	+/− 17	81	+/− 16	<0.001	78	+/− 17
*n*	1,440		1,253		799		1,309			3,671	
Arterial hypertension *	893	60.0	966	74.0	217	26.6	571	42.6	< 0.001	2,647	53.5
Dyslipidaemia *	706	47.5	637	48.8	266	32.6	579	43.2	< 0.001	2,188	44.2
Diabetes Mellitus *	402	27.0	372	28.5	70	8.6	245	18.3	< 0.001	1,089	22.0
Current smoking *	357	24.0	64	6.4	595	73.0	764	57.1	< 0.001	1,780	36.0
Comorbidity *	475	31.9	543	41.6	136	16.7	259	19.3	< 0.001	1,413	28.6
Initial presentation (first MI event) *	905	60.8	583	44.7	682	83.7	988	73.8	< 0.001	3,158	63.8
Treatments in progress at the time of AMI											
Proton pump inhibitor*	399	26.8	421	32.3	94	11.5	237	17.7	< 0.001	1,151	23.3
Diuretics *	324	21.8	431	33	45	5.5	148	11.1	< 0.001	948	19.2
Betablocker *	516	34.7	507	38.9	150	18.4	324	24.2	< 0.001	1,497	30.3
Calcium channel blocker *	271	18.2	354	27.1	41	5.0	139	10.4	< 0.001	805	16.3
ACE inhibitor *	327	22.0	312	23.9	110	13.5	235	17.6	< 0.001	984	19.9
ARB *	264	17.7	284	21.8	44	5.4	136	10.2	< 0.001	728	14.7
Statin *	570	38.3	524	40.2	164	20.1	379	28.3	< 0.001	1,637	33.1
Levothyroxyn *	88	5.9	118	9.0	14	1.7	45	3.4	< 0.001	265	5.4
Anti-inflammatory drug *	30	2.0	38	2.9	7	0.9	17	1.3	0.002	92	1.9
Opioïds *	47	3.2	64	4.9	17	2.1	38	2.8	0.002	166	3.4
Antiepileptic *	56	3.8	66	5.1	18	2.2	37	2.8	0.001	177	3.6
Anxiolytics *	120	8.1	143	11.0	57	7.0	83	6.2	< 0.001	403	8.1
Sleeping pills *	54	3.6	94	7.2	16	2.0	33	2.5	< 0.001	197	4.0
Antidepressant *	95	6.4	95	7.3	39	4.8	66	4.9	0.028	295	6.0
Clinical presentation											
Diagnosis at admission *									<0.001		
STEMI	697	46.8	512	39.2	504	61.8	745	55.6		2,458	49.7
NSTEMI	791	53.2	793	60.8	311	38.2	594	44.4		2,489	50.3
If STEMI											
Anterior	302/670	45.1	231/472	48.9	207/498	41.6	303/729	41.6	0.047	1,043/2,369	44.0
Inferior / Posterior	344/670	51.3	220/472	46.6	255/498	51.2	402/729	55.1	0.038	1,221/2,369	51.5
Lateral	97/670	14.5	73/472	15.5	99/498	19.9	106/729	14.5	0.046	375/2,369	15.8
Undetermined	21/670	3.1	21/472	4.5	15/498	3.0	17/729	2.3	0.234	74/2,369	3.1
Typical chest pain	1,179	81.6	943	75.3	709	88.0	1,107	83.8	< 0.001	3,938	81.6
Heart failure/acute pulmonary edema	84	5.9	158	12.8	11	1.4	30	2.3	< 0.001	283	5.9
Cardiac arrest	21	1.5	17	1.4	19	2.4	31	2.4	0.122	88	1.8
Syncope	50	3.5	39	3.1	22	2.8	50	3.8	0.586	161	3.4
Killip = III / IV	53	3.9	93	7.7	13	1.7	29	2.3	< 0.001	188	4.1
Heart rate (bpm)	77	+/− 18	80	+/− 20	79	+/− 16	78	+/− 17	< 0.001	78	+/−18
Systolic blood pressure (mmHg)	142	+/− 27	143	+/− 28	136	+/− 25	138	+/− 27	< 0.001	140	+/− 27
Diastolic blood pressure (mmHg)	80	+/− 16	77	+/− 15	84	+/− 16	83	+/− 16	< 0.001	80	+/− 16
Biological presentation											
LDL-cholesterol (mmol/L)									<0.001		
Tertile 1 (<1.34)	428	28.8	377	28.9	155	19.0	307	22.9		1,267	25.6
Tertile 2 (1.34–2.75)	398	26.7	314	24.1	221	27.1	358	26.7		1,291	26.1
Tertile 3 (> 2.75)	352	23.7	236	18.1	279	34.2	427	31.9		1,294	26.2
Unknown	310	20.8	378	29.0	160	19.6	247	18.4		1,095	22.1
HDL-cholesterol (mmol/L)									<0.001		
Tertile 1 (< 0,54)	380	25.5	278	21.3	265	32.5	392	29.3		1,315	26.6
Tertile 2 (0.54–1.04)	382	25.7	289	22.1	235	28.8	397	29.6		1,303	26.3
Tertile 3 (> 1.04)	428	28.8	371	28.4	170	20.9	328	24.5		1,297	26.2
Unknown	298	20.0	367	28.1	145	17.8	222	16.6		1,032	20.9
Triglycerides (mmol/L)									<0.001		
Tertile 1 (< 1.17)	252	16.9	255	19.5	114	14.0	148	11.1		769	15.5
Tertile 2 (1.17–1.76)	247	16.6	184	14.1	115	14.1	226	16.9		772	15.6
Tertile 3 (> 1.76)	198	13.3	104	8.0	179	22.0	290	21.7		771	15.6
Unknown	791	53.2	762	58.4	407	49.9	675	50.4		2,635	53.3
C Reactive Protein (mg/L) *									<0.001		
Tertile 1 (< 3.1)	435	29.2	336	25.7	213	26.1	437	32.6		1,421	28.7
Tertile 2 (3.1–8.6)	315	21.2	280	21.5	215	26.4	336	25.1		1,146	23.2
Tertile 3 (> 8.6)	338	22.7	455	34.9	176	21.6	298	22.3		1,267	25.6
Unknown	400	26.9	234	17.9	211	25.9	268	20.0		1,113	22.5
Leucocytes (10^3^/mm^3^)									<0.001		
Tertile 1 (< 8.4)	545	36.6	488	37.4	155	19.0	385	28.8		1,573	31.8
Tertile 2 (8.4–11.4)	477	32.1	421	32.3	239	29.3	416	31.1		1,553	31.4
Tertile 3 (> 11.4)	397	26.7	347	26.6	365	44.8	458	34.2		1,567	31.7
Unknown	69	4.6	49	3.8	56	6.9	80	6.0		254	5.1
Red cells (10^6^/mm^3^)									<0.001		
Tertile 1 (≤ 4.4)	490	32.9	662	50.7	122	15.0	273	20.4		1,547	31.3
Tertile 2 (4.41–4.9)	476	32.0	367	28.1	258	31.7	454	33.9		1,555	31.4
Tertile 3 (> 4.9)	426	28.6	214	16.4	360	44.2	521	38.9		1,521	30.7
Unknown	96	6.5	62	4.8	75	9.2	91	6.8		324	6.5
Platelets (10^3^/mm^3^)									<0.001		
Tertile 1 (< 209)	540	36.3	500	38.3	162	19.9	385	28.8		1,587	32.1
Tertile 2 (209–261)	497	33.4	404	31.0	266	32.6	421	31.4		1,588	32.1
Tertile 3 (> 261)	387	26.0	362	27.7	349	42.8	470	35.1		1,568	31.7
Unknown	64	4.3	39	3.0	38	4.7	63	4.7		204	4.1
Hemoglobin (g/dL)									<0.001		
Tertile 1 (< 13.6)	535	36.0	739	56.6	136	16.7	285	21.3		1,695	34.3
Tertile 2 (13.6–14.9)	464	31.2	342	26.2	246	30.2	443	33.1		1,495	30.2
Tertile 3 (≥ 15)	437	29.4	194	14.9	402	49.3	558	41.7		1,591	32.2
Unknown	52	3.5	30	2.3	31	3.8	53	4.0		166	3.4
Glycaemia (mmol/L)									<0.001		
Tertile 1 (< 4.63)	413	27.8	354	27.1	211	25.9	394	29.4		1,372	27.7
Tertile 2 (4.64–6.70)	417	28.0	334	25.6	270	33.1	402	30.0		1,423	28.8
Tertile 3 (> 6.70)	446	30.0	404	31.0	197	24.2	348	26.0		1,395	28.2
Unknown	212	14.2	213	16.3	137	16.8	195	14.6		757	15.3
Creatinine (μmol/L) *									<0.001		
Tertile 1 (< 72.57)	425	28.6	339	26.0	323	39.6	519	38.8		1,606	32.5
Tertile 2 (72.57–91.00)	519	34.9	321	24.6	303	37.2	482	36.0		1,625	32.8
Tertile 3 (> 91.00)	509	34.2	606	46.4	149	18.3	287	21.4		1,551	31.4
Unknown	35	2.4	39	3.0	40	4.9	51	3.8		165	3.3

*Variables marked with an asterisk (*) Were included in the unsupervised clustering analysis. All these variables were available at admission or reflected pre-existing patient characteristics. **mean +/− standard deviation. ACE, Angiotensin-converting enzyme; ARB, Angiotensin II Receptor Blocker; BMI, Body Mass Index; MI, Myocardial infarction.

### Early hospital management

After hospital admission, coronary angiography was almost systematically performed except in Cluster 2 (92% vs. > 98% in the other clusters, *p* < 0.001). Multivessel disease was more frequently observed in Clusters 1 and 2 (59.6 and 63.2%) than in Clusters 3 and 4 (38.1 and 49.4%, *p* < 0.001). Revascularization was less frequently observed in Cluster 2 (71%) than in other clusters (>80%, *p* < 0.001). Regarding medical treatments, aspirin was widely and equally prescribed in all groups, but other medications such as betablockers, angiotensin converting enzyme inhibitors (ACEI) or statins were significantly less frequently used in Clusters 1 and 2.

### In-hospital outcome

Complications were more frequently observed in Clusters 1 and 2: atrioventricular block (*p* < 0.001), pacemaker implantation (*p* < 0.001), left intraventricular thrombus (*p* < 0.001) or mechanical complication (*p* < 0.001). These two groups received more frequently a life support assist device (intra-aortic balloon pump (IABP), ventricular assist, mechanical ventilation) (*p* < 0.001). Furthermore, minor or major bleeding (*p* = 0.001) or transfusion (p = 0.001) were also more frequently observed in Clusters 1 and 2.

In hospital mortality was higher in Clusters 1 (1.8%) and 2 (4.9%) than in Clusters 3 and 4 (0.4 and 0.7% respectively) (*p* < 0.001).

### Mortality and prognostic factors

**Figure 2:**
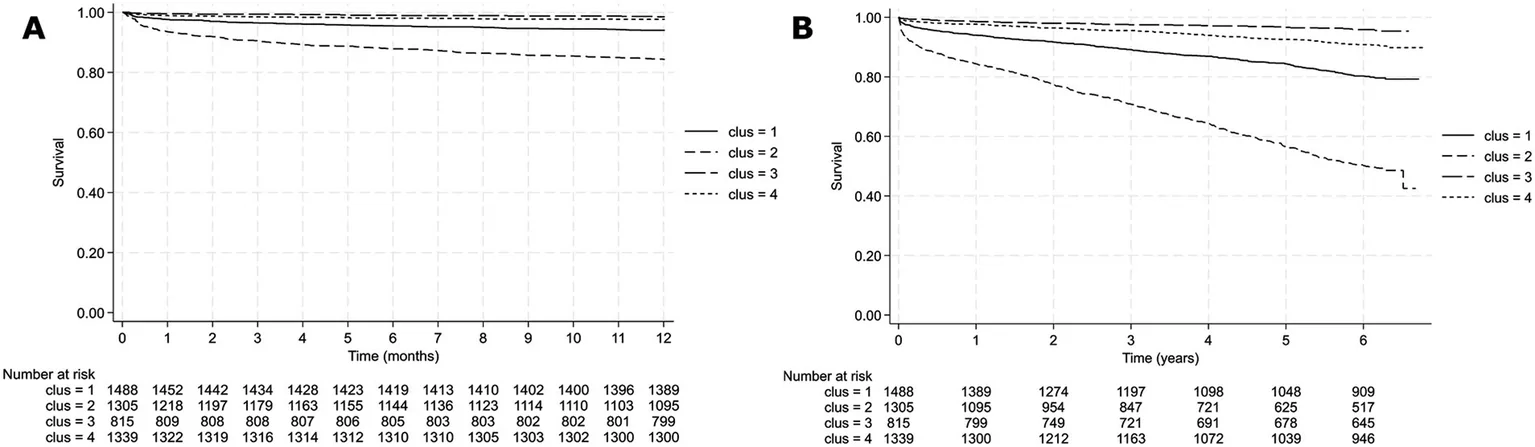
Kaplan–Meier survival curves according to cluster membership. **(A)** One-year survival. **(B)** Long-term survival (median follow-up 6.18 years).

The median follow-up duration for these 4,947 patients was 6.18 years, corresponding to a total of 24,247 person-years of follow-up and 1,037 deaths. One-year mortality differed markedly across clusters, with the highest mortality observed in Cluster 2 and the lowest in Cluster 3 (Table 2; Figure 2A). Similar differences were observed over the entire follow-up period, with persistent separation of Kaplan–Meier survival curves up to 6.8 years (Figure 2B; log-rank *p* < 0.001).

**Table 2:** In-hospital management, complications and outcomes according to cluster.

	Cluster 1 (*n* = 1,488)		Cluster 2 (*n* = 1,305)		Cluster 3 (*n* = 815)		Cluster 4 (*n* = 1,339)		*p*	Overall (*n* = 4,947)	
	*n*	%	*n*	%	*n*	%	*n*	%		*n*	%
In-hospital management											
Any assistance *	190	12.8	209	16.1	55	6.8	135	10.2	< 0.001	589	12.0
Coronarography performed	1,464	98.4	1,203	92.2	807	99.0	1,332	99.5	< 0.001	4,806	97.1
If coronarography performed											
GPIIbIIIa agonists	170	11.9	80	6.8	137	17.4	198	15.2	< 0.001	585	12.5
Radial access	1,274	87.9	1,000	84.2	728	90.6	1,203	91.1	< 0.001	4,205	88.3
Initial LVEF (%)	50	+/− 11	49	+/− 12	53	+/− 10	53	+/− 11	< 0.001	51	+/− 11
Result									0.024		
Normal	74	5.1	38	3.2	31	3.9	41	3.1		184	3.8
Abnormal	1,388	94.9	1,162	96.8	774	96.1	1,288	96.9		4,612	96.2
If abnormal (vessels with >50% stenosis)									< 0.001		
Mono or stenosis < 50%	538	40.4	408	36.9	446	61.9	621	50.6		2,013	45.9
Bi	473	35.5	354	32.0	187	26.0	390	31.8		1,404	32.0
Tri	321	24.1	345	31.2	87	12.1	216	17.6		969	22.1
PCI performed	1,141	76.7	898	68.9	684	84.1	1,137	85.0	< 0.001	3,860	78.1
If PCI performed											
Culprit lesion treated	1,114	97.8	869	97.2	673	98.8	1,115	98.5	0.071	3,771	98.0
Intracoronary thrombus	194	17.7	109	12.7	171	25.7	228	20.7	< 0.001	702	18.9
Other lesion treated	222	19.6	212	23.8	90	13.4	166	14.7	< 0.001	690	18.0
Stent(s)	1,064	93.3	829	92.6	647	95.0	1,090	95.9	0.007	3,630	94.2
If stent											
BMS	139	13.2	175	21.3	72	11.3	136	12.6	< 0.001	522	13.7
DES	949	89.2	687	82.9	583	90.1	982	90.1	< 0.001	3,201	83.1
if DES, total length (mm)									< 0.001		
Tertile 1	304	33.3	199	29.9	220	39.6	335	35.7		1,058	34.5
Tertile 2	296	32.5	223	33.5	192	34.5	296	31.6		1,007	32.8
Tertile 3	312	34.2	243	36.5	144	25.9	307	32.7		1,006	32.8
Thromboaspiration	210	18.9	104	11.9	160	23.9	236	21.1	< 0.001	710	18.8
Final TIMI III	948	94.9	757	95.3	581	96.4	984	97.7	0.007	3,270	96.1
PCI success	1,106	97.1	859	96.0	667	98.1	1,107	97.7	0.047	3,739	97.2
Complete revascularisation	758	71.9	573	66.8	506	78.5	812	74.8	< 0.001	2,649	72.7
If PCI performed within 48 h											
Time to balloon									< 0.001		
< 3 h30	202	24.2	106	17.3	169	28.6	286	30.8		763	25.4
3 h30 - 8 h	187	22.4	156	25.4	155	26.2	216	23.2		714	23.8
8 h - 22 h	225	27.0	165	26.9	140	23.7	214	23.0		744	24.8
22 h - 48 h	221	26.5	221	30.5	128	21.6	214	23.0		784	26.1
CABG performed	79	5.3	35	2.7	7	0.9	29	2.2	< 0.001	150	3.0
Revascularisation performed (PCI or CABG)	1,208	81.2	931	71.3	691	84.8	1,164	86.9	< 0.001	3,994	80.7
Medical treatments used in the first 48 h											
Antiplatelet drugs (aspirin and/or P2Y12 inhibitors)	1,458	98.0	1,268	97.2	803	98.5	1,324	98.9	0.01	4,853	98.1
Proton pump inhibitor	919	61.8	813	62.3	443	54.4	813	60.7	0.001	2,988	60.4
Diuretics	445	29.9	597	45.8	95	11.7	239	17.9	< 0.001	1,376	27.8
Betablocker	1,129	75.9	897	68.7	615	75.5	1,031	77.0	< 0.001	3,672	74.2
Calcium channel blocker	440	29.6	408	31.3	164	20.1	318	23.8	< 0.001	1,330	26.9
ACE inhibitor	765	51.4	542	41.5	449	55.1	755	56.4	< 0.001	2,511	50.8
ARB	179	12.0	204	15.6	37	4.5	93	7.0	< 0.001	513	10.4
ACE inhibitor or ARB	928	62.4	736	56.4	482	59.1	838	62.6	0.002	2,984	60.3
Statin	1,212	81.5	989	75.8	676	82.9	1,152	86.0	< 0.001	4,029	81.4
In-hospital outcomes											
Death	27	1.8	64	4.9	3	0.4	9	0.7	< 0.001	103	2.1
Ischemic recurrence **	35	2.4	31	2.4	11	1.4	21	1.6	0.175	98	2.0
Atrioventricular block II/III appearance	28	1.9	41	3.2	5	0.6	14	1.1	0.006	88	1.8
Mechanical complication ***	21	1.4	40	3.1	3	0.4	10	0.8	< 0.001	74	1.5
Left intraventricular thrombus	8	0.5	10	0.8	5	0.6	8	0.6	0.894	31	0.6
Non-compressive pericardial effusion	20	1.4	16	1.2	2	0.3	8	0.6	0.021	46	0.9
Stroke / TIA	7	0.5	16	1.2	1	0.1	6	0.5	0.01	30	0.6
Major bleeding	8	0.5	9	0.7	1	0.1	3	0.2	0.139	21	0.4
Minor or major bleeding	13	0.9	18	1.4	1	0.1	4	0.3	0.001	36	0.7
Transfusion	55	3.7	80	6.1	7	0.9	13	1.0	< 0.001	155	3.1
Pacemaker implantation	11	0.7	18	1.4	0	0.0	1	0.1	< 0.001	30	0.6
Defibrillator implantation	14	1.0	10	0.8	4	0.5	7	0.5	0.489	35	0.7
Intensive care stay	41	2.8	30	2.3	11	1.4	21	1.6	0.055	103	2.1
Length of hospital stay (days) ****	6	4–9	7	5–11	5	4–7	5	4–7	< 0.001		
One year mortality											
Cumulative probability of death at 1y (%)	6.0 [4.9 – 7.3] *****		15.7 [13.8–17.8]		1.5 [0.8–2.6]		2.3 [1.6–3.3]		< 0.001	6.8 [6.1–7.5]	

*CPBIA, ventricular assistance (ECMO, Impella…), Assisted or non-assisted ventilation. **Electric signs of ischaemia, recurrence of necrosis or stent thrombosis. ***Mitral insufficiency > Grade II, free wall rupture, ventricular septal defect. ****Median and interquartile range. *****With 95% confidence interval. ACE, Angiotensin-converting enzyme; ARB, Angiotensin II Receptor Blocker; BMS, Bare Metal Stent; CABG, Coronary Artery Bypass Graft; DES, Drug Eluting Stent; LVEF, Left Ventricular Ejection Fraction; PCI, Percutaneous Coronary Intervention; TIA, Transcient Ischemic Accident.

Multivariate analyses were conducted separately in Clusters 1 and 2 to identify independent predictors of 1 year mortality. Because of the limited number of one-year mortality events in Clusters 3 and 4 (12 and 31 events, respectively), multivariable Cox analyses were not performed in Clusters 3 and 4.

**Figure 3:**
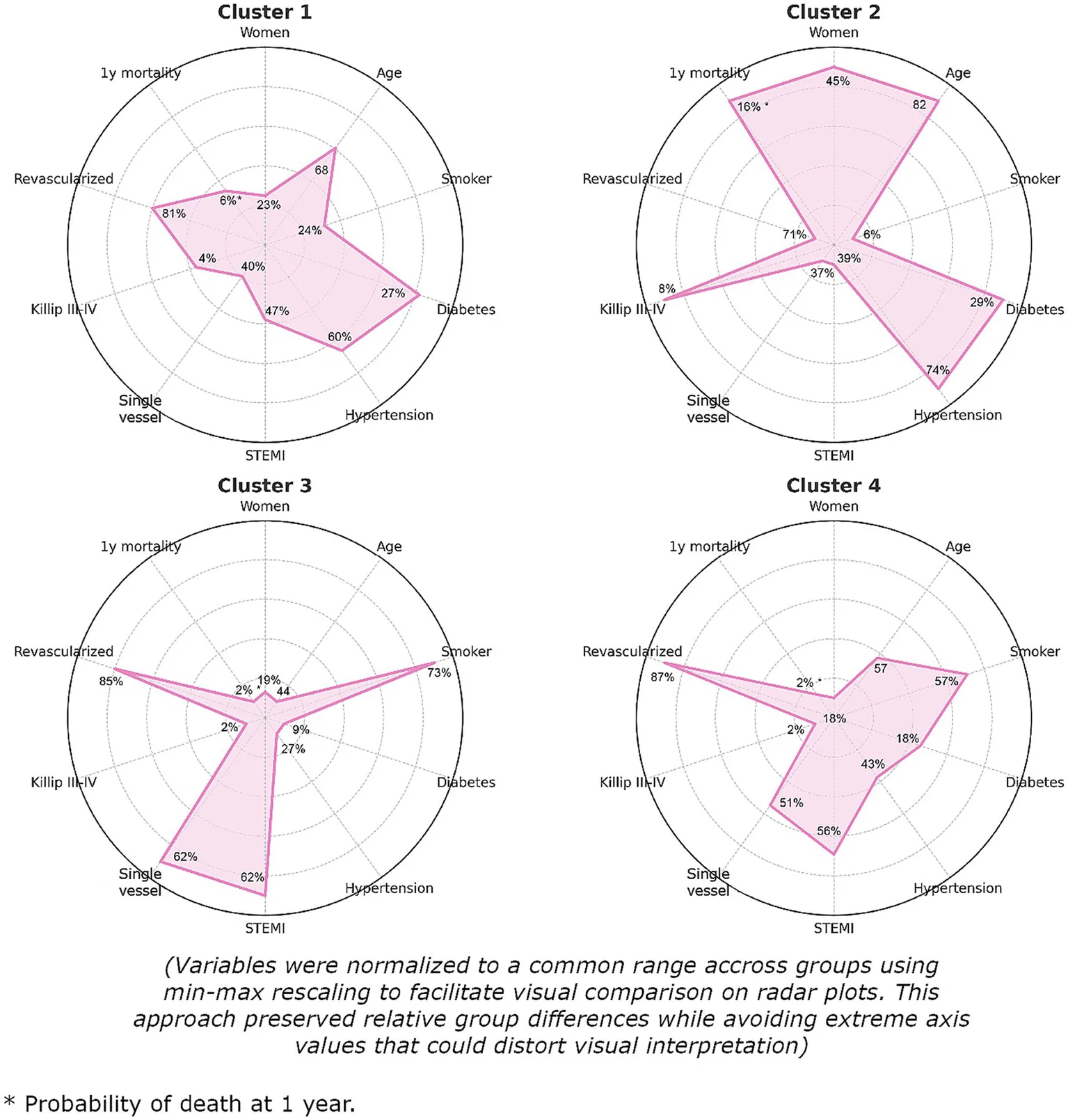
Graphical summary of baseline characteristics, revascularisation therapy and 1 year mortality rate by patient cluster.

Multivariable analyses identified distinct prognostic factors in Clusters 1 and 2 (Table 3). While cardiac arrest at presentation was a strong predictor of mortality in both clusters, several other predictors differed between Cluster 1 and Cluster 2. Myocardial revascularization was associated with lower mortality in Cluster 1 [HR = 0.36 (0.22–0.59), *p* < 0.001] but not in Cluster 2, whereas statin use was associated with lower mortality solely in Cluster 2 [HR = 0.65 (0.48–0.88), *p* = 0.005]. Figure 3 provides a graphical overview of the key differences in baseline characteristics, revascularization strategies, and one-year mortality across the four clusters.

**Table 3:** Multivariate Cox models for 1-year mortality: Independent predictors in Clusters 1 and 2.

	Cluster 1 (*n* = 1,488)			Cluster 2 (*n* = 1,305)		
	HR	95% CI	*p*	HR	95% CI	*p*
Age (years)	1.09	1.03–1.16	0.002	1.04	1.01–1.07	0.003
Diabetes Mellitus	1.70	1.10–2.62	0.017			
Comorbidity	1.95	1.26–3.02	0.003	1.66	1.24–2.21	0.001
Heart failure/APE						
Cardiac arrest						
No	1.00	(ref)		1.00	(ref)	
Yes	5.72	2.42–13.53	< 0.001	5.45	2.54–11.69	< 0.001
*Unknown*	1.14	0.41–3.15	0.805	1.15	0.64–2.04	0.646
Killip						
I - II	1.00	(ref)		1.00	(ref)	
III - IV	2.74	1.49–5.02	0.001	1.66	1.11–2.50	0.014
*Unknown*	0.75	0.33–1.69	0.491	0.91	0.54–1.54	0.725
Heart rate (bpm)						
< 70				1.00	(ref)	
≥ 70				1.51	1.03–2.20	0.035
*Unknown*				0.60	0.15–2.46	0.476
Systolic Blood Pressure (mmHg)						
< 150	1.00	(ref)		1.00	(ref)	
≥ 150	0.38	0.22–0.68	0.001	0.63	0.46–0.87	0.005
*Unknown*	1.52	0.68–3.38	0.307	1.52	0.37–6.27	0.560
C Reactive Protein (mg/L)						
< 5	1.00	(ref)		1.00	(ref)	
≥ 5	2.32	1.28–4.20	0.006	1.60	1.08–2.36	0.019
*Unknown*	1.61	0.81–3.19	0.173	1.32	0.80–2.18	0.273
Leucocytes (10^3^/mm^3^)						
< 12				1.00	(ref)	
≥ 12				1.50	1.11–2.04	0.009
*Unknown*				1.30	0.57–2.94	0.537
Red cells (10^6^/mm^3^)						
Tertile 1 (≤ 4.4)	3.75	1.84–7.66	< 0.001			
Tertile 2 (4.41 - 4.9)	1.00	(ref)				
Tertile 3 (> 4.9)	2.83	1.25–6.38	0.012			
*Unknown*	3.02	1.12–8.16	0.029			
Hemoglobin (g/dL)						
< 13				1.00	(ref)	
≥ 13				0.59	0.44–0.80	0.001
*Unknown*				0.26	0.06–1.20	0.085
Creatinine (μmol/L)						
< 100				1.00	(ref)	
≥ 100				1.70	1.25–2.30	0.001
*Unknown*				1.93	0.80–4.65	0.144
Initial LVEF (%)						
< 40				1.00	(ref)	
≥ 40				0.53	0.37–0.75	< 0,001
*Unknown*				0.89	0.58–1.36	0.596
Disease extent						
1 vessel	1.00	(ref)		1.00	(ref)	
2 vessels	2.15	1.24–3.72	0.007			
3 vessels				1.65	1.19–2.28	0.002
*Unknown*	0.99	0.44–2.25	0.984	1.51	0.99–2.30	0.054
PCI or CABG performed *(forced variable)*	0.36	0.22–0.59	< 0.001	0.96	0.67–1.36	0.800
Antiplatelet agents *in the first 48 h*						
Diuretics *in the first 48 h*				1.63	1.19–2.24	0.002
Calcium channel blocker *in the first 48 h*						
Statin *in the first 48 h*				0.65	0.48–0.88	0.005

APE, Acute Pumonary Edema; CABG, Coronary Artery Bypass Graft; CI, Confidence interval. HR: Hazard Ratio. LVEF: Left Ventricular Ejection Fraction. PCI: Percutaneous Coronary Intervention.

## Discussion

In this study, unsupervised clustering analysis identified four phenotypes among patients admitted for AMI in the FAST-MI 2015 cohort, in patients living in a Western European Country. These clusters differed markedly in terms of age, comorbidities, cardiovascular risk profiles, AMI presentation, in-hospital management, and outcomes. Interestingly, these four groups appear to correspond to phenotypes that clinicians commonly encounter in their daily practice.

Cluster 1 appeared to pool together patients corresponding to a profile of a “typical older and intermediate risk coronary patient,” composed predominantly of men, with a mean age of 68 years, carrying cardiovascular risk factors (hypertension, diabetes, dyslipidemia) but with few non-cardiovascular comorbidities. This was an intermediate-risk group, well managed, with a relatively favorable one-year prognosis. Cluster 2 seemed to correspond to a profile of an “elderly polymorbid patient,” with a mean age of 82 years. Women were over represented in this group as compared to other groups. Cardiovascular risk factors (hypertension, dyslipidemia, diabetes) were very common, and chronic use of medications before the event (betablockers, diuretics, statins, psychotropic drugs…) and comorbidities were multiple. In this cluster, NSTEMI presentations were more frequent, coronary lesions more diffuse, and the initial clinical presentation more severe (Killip >2: 7.7%). These patients were less likely to undergo invasive revascularization procedures, and even to receive the recommended medical treatments, and their one-year prognosis was the poorest among the four clusters, likely due to age and overall frailty. Cluster 3 patients seemed to match the profile of “young smokers without prior medical history,” few pre-existing treatments and few comorbidities, entering in CAD generally through STEMI, for most with typical symptoms and usually with single vessel disease. Despite the initial severity of the AMI, the prognosis in this group was excellent, likely due to young age and the absence of comorbidities, effective invasive management, and high prescription rates of recommended medications. Cluster 4 patients were very similar to those in Cluster 3 but were older and exhibited a more metabolic profile. Although patients in this group, like those in Cluster 3, were predominantly male and often active smokers, they were also more frequently affected by diabetes, hypertension, and dyslipidemia. They generally underwent invasive revascularization, experienced few in-hospital complications, and the one-year prognosis was very good. This favorable outcome was likely related to the absence of severe comorbidities.

In Cluster 2 - the oldest, most comorbid and predominantly NSTEMI phenotype - revascularization was not associated with lower one-year mortality (HR 0.96, 95% CI 0.67–1.36), in contrast to the marked benefit observed in Cluster 1 (HR 0.36, 95% CI 0.22–0.59). Several non-exclusive explanations should be considered. First, this observational estimate is susceptible to confounding by indication operating in opposite directions: frailer patients may have been declined invasive management (which would bias toward an apparent benefit), whereas a residual effect linked to “healthier patients” selected for procedure may also be present; the net result is uninterpretable as a causal effect. Second, the attenuation may be genuine: in elderly, frail and polymorbid patients, competing non-cardiovascular mortality, diffuse and calcified coronary disease, and procedural risk can dilute the prognostic benefit of revascularization, consistent with emerging evidence from geriatric cardiology and from randomized data in older NSTEMI populations (Sanchis et al., 2023). Third, the finding should not be read as a recommendation against invasive management in this group; rather, it underscores that the benefit–risk balance is narrower and more individualized in frail patients, and that treatment decisions should integrate frailty, life expectancy and patient preference. This hypothesis is hypothesis-generating.

Beyond the descriptive identification of patient phenotypes, the potential value of this approach lies in its integration of multiple correlated dimensions into a single clinically recognizable profile rather than treating age, sex, comorbidity and presentation as separate axes - which is closer to how clinicians actually reason at the bedside. Importantly, the clusters carry information about treatment-outcome relationships that conventional characteristics do not make explicit: the differential association of revascularization with survival (beneficial in Cluster 1, null in Cluster 2) emerges at the level of the phenotype.

Our results are consistent with prior works in cardiovascular medicine using clustering to explore patient’s heterogeneity in cardiovascular disease and particularly in AMI. Thus, in the ADDICT-ICCU study, Hamzi et al. (2024) identified four distinct phenotypes among patients hospitalized in intensive cardiac care units (ICCU), including a group of young STEMI patients with relatively favorable prognosis and a frailer group with heart failure and preserved ejection fraction, mirroring our Clusters 3/4 and 2, respectively. Chunta and Miller (2024) applied clustering specifically to STEMI patients and identified profiles including one with « behavioral risks », high rate of invasive revascularization and favorable outcomes, and another gathering patients with diabetes and end-organ damages, less frequent use of invasive revascularization, and worse outcomes. Other studies conducted in ICCU outside of an AMI also echo the results observed in our study. Sharma et al. (2022) used clustering in patients with diabetes and atherosclerotic cardiovascular disease, identifying distinct phenotypes that could be compared to those we observed in this study, finding in particular a duality between an elderly high-risk group with comorbidities and a lower-risk group composed of younger patients with a less advanced disease. Similarly, Holtrop et al. (2025) used latent class analysis in patients with stable CAD and isolated phenotypes comparable to those we observed, with, in particular, a group of elderly polyvascular patients with a poor prognosis, and a group of young patients, with a high frequency of smoking but also dyslipidemia and history of MI. Finally, even in patients with normal cardiac perfusion (Miller et al., 2024), distinct phenotypes with very different prognosis can be identified, suggesting that standard diagnostic categories like normal imaging may hide high-risk subgroups, which could be better characterized by clustering approaches. These findings across various clinical contexts converge toward the same conclusion that clustering approaches could help discover reproducible and clinically meaningful subgroups of patients. Our findings in the FAST-MI 2015 study add to this growing body of evidence that, in the context of AMI, unsupervised clustering uncovers distinct phenotypes with radically different management patterns and prognosis.

Several unsupervised clustering methods are available, each with its strengths and limitations. Ward’s hierarchical clustering was chosen for its interpretability and ability to generate compact, balanced clusters without requiring prior specification of the number of groups. Other techniques, such as k-means, latent class analysis, or Gaussian mixture models, may offer complementary insights but often require additional assumptions, particularly a predefined number of clusters, or are less suited to mixed-type clinical data. In our study, a sensitivity analysis using k-means clustering yielded a comparable four-cluster solution, with a high degree of overlap in patient assignment and similar clinical profiles across groups, supporting the robustness of the 4 phenotypes identified using Ward’s hierarchical clustering.

Although the two-cluster solution yielded the highest silhouette coefficient, it merged clinically distinct patient profiles into two broad groups, thus masking important differences in age, smoking status, metabolic profile, and cardiovascular risk burden. So, even if silhouette coefficients were slightly higher for simpler partitions (k = 2 and k = 3), inspection of the hierarchical structure showed that the transition from three to four clusters corresponded to a subdivision of the youngest patient group into two clinically distinct phenotypes, whereas the two older phenotypes remained unchanged. The four-cluster solution was therefore retained because it provided additional clinical discrimination while preserving a satisfactory level of cluster cohesion.

Relatively free from preconceived assumptions, unsupervised clustering techniques are likely to “let the data speak,” revealing distinct patient profiles that would not be apparent using conventional subgroup analyses or regression-based stratifications.

However, while unsupervised clustering enables the identification of data-driven patient profiles without predefined clinical assumptions, several methodological limitations must be acknowledged. First, the clustering output is dependent on which variables were selected to conduct the analysis. In our study, only baseline variables available at admission or reflecting pre-existing patient characteristics were included in the clustering procedure, but important clinical dimensions - such as frailty, socioeconomic status, or specific biomarkers - may be missing, potentially biasing the resulting clusters obtained. Age emerged as the strongest variable associated with cluster membership. This finding is not unexpected given the central role of age in the epidemiology, presentation, management and prognosis of AMI. Nevertheless, it indicates that the identified phenotypes are partly driven by age-related differences, which should be considered when interpreting the results. Importantly, age alone did not fully account for cluster separation, as smoking status, cardiovascular risk profile, comorbidity burden and clinical presentation also contributed substantially to phenotypes definition. Secondly, given the observational nature of the study and the potential for non-random missingness, we chose to retain patients with missing values, notably with missing values for laboratory results, by creating a separate « unknown » category. This approach may introduce bias while this « unknown » category may reflect underlying patient severity or care pathways. In addition, patients excluded from the clustering analysis because of missing data were older, more frequently female, more likely to present with STEMI and higher Killip class, and experienced higher one-year mortality than included patients. Therefore, the clustering analysis was conducted in a slightly healthier population, which may have influenced the distribution of phenotypes and could limit the generalizability of the findings to the highest-risk patients. Thirdly, the clusters identified may be cohort-specific and not generalizable to other populations without external validation. FAST-MI 2015 reflects clinical practice and patient characteristics from the mid-2010s. Since then, substantial changes in AMI management and secondary prevention strategies have occurred, which may limit the direct applicability of these findings to more contemporary populations. In addition, FAST-MI included patients admitted to ICCU in a Western European setting; our results therefore may not apply to patients who would have died before hospital admission, and may not be generalizable to other geographic or healthcare contexts. Additionaly, although the identified phenotypes were clinically coherent and associated with markedly different outcomes, we did not formally compare their prognostic performance with established risk scores such as GRACE or TIMI, which remains an important next step. Finally, although the identified phenotypes were clinically interpretable, this clustering approach does not provide a directly deployable tool for assigning new patients to a cluster. External validation and development of a simplified classifier capable of reproducing cluster assignment in independent populations would be necessary before these phenotypes could be used in clinical practice.

Thus, while clustering holds promise, its findings must be interpreted with caution and ideally confirmed in others independent datasets.

## Conclusion

In this nationwide registry of patients with AMI, unsupervised clustering identified four clinically distinct phenotypes with meaningful differences in presentation, early management, and one-year prognosis. Less intensive management strategies were observed in older and more vulnerable patients, regardless of ST-segment classification, whereas younger patients, mostly presenting with STEMI, more often received guideline-recommended invasive management and had better outcomes. These findings underscore the heterogeneity hidden within conventional diagnostic categories and highlight the potential of machine learning methods to reveal clinically relevant subgroups. From a clinical perspective, this phenotypic approach may support more informed clinical decision-making, enhance risk stratification, and could contribute to more personalized management strategies.

## Data Availability

The datasets presented in this article are not readily available because they contain individual-level clinical data from the FAST-MI 2015 registry. The data are owned and managed by the French Society of Cardiology and are subject to ethical, legal, and governance restrictions. Requests to access the datasets should be directed to contact@sfcardio.fr.

## References

[ref1] Banerjee A. Chen S. Fatemifar G. Zeina M. Lumbers R. T. Mielke J. . (2021). Machine learning for subtype definition and risk prediction in heart failure, acute coronary syndromes and atrial fibrillation: systematic review of validity and clinical utility. BMC Med. 19:85. doi: 10.1186/s12916-021-01940-7, 33820530 PMC8022365

[ref2] Bouisset F. Gerbaud E. Bataille V. Coste P. Puymirat E. Belle L. . (2021). Percutaneous myocardial revascularization in late-presenting patients with STEMI. J. Am. Coll. Cardiol. 78, 1291–1305. doi: 10.1016/j.jacc.2021.07.039, 34556314

[ref3] Byrne R. A. Rossello X. Coughlan J. J. Barbato E. Berry C. Chieffo A. . (2023). 2023 ESC guidelines for the management of acute coronary syndromes. Eur. Heart J. 44, 3720–3826. doi: 10.1093/eurheartj/ehad191, 37622654

[ref4] Chunta A. Miller R. J. H. (2024). Uncovering STEMI patient phenotypes using unsupervised machine learning. Int. J. Cardiol. 413:132346. doi: 10.1016/j.ijcard.2024.132346, 38992808

[ref5] Hamzi K. Gall E. Roubille F. Trimaille A. Elbaz M. El Ouahidi A. . (2024). Phenotypic clustering of patients hospitalized in intensive cardiac care units: insights from the ADDICT-ICCU study. Arch. Cardiovasc. Dis. 117, 392–401. doi: 10.1016/j.acvd.2024.03.004, 38834393

[ref6] Holtrop J. Lim C. E. Uijl A. Ueda P. Jernberg T. Van der Meer M. G. . (2025). Identifying clinical phenotype clusters in patients with coronary artery disease. Heart:heartjnl-2025-325740. doi: 10.1136/heartjnl-2025-325740, 40451277

[ref7] Jacquemyn X. Chinni B. K. Barnes B. T. Rao S. Kutty S. Manlhiot C. (2024). Unsupervised machine learning identifies distinct phenotypes in cardiac complications of pediatric patients treated with anthracyclines. Cardiooncology. 10:74. doi: 10.1186/s40959-024-00276-4, 39468669 PMC11514752

[ref8] Loftus T. J. Shickel B. Balch J. A. Tighe P. J. Abbott K. L. Fazzone B. . (2022). Phenotype clustering in health care: a narrative review for clinicians. Front Artif Intell. 5:842306. doi: 10.3389/frai.2022.842306, 36034597 PMC9411746

[ref9] Mezilis N. Dardas P. Ninios V. Tsikaderis D. (2010). Rotablation in the drug eluting era: immediate and long-term results from a single center experience. J. Interv. Cardiol. 23, 249–253. doi: 10.1111/j.1540-8183.2010.00542.x, 20459456

[ref10] Miller R. J. H. Bednarski B. P. Pieszko K. Kwiecinski J. Williams M. C. Shanbhag A. . (2024). Clinical phenotypes among patients with normal cardiac perfusion using unsupervised learning: a retrospective observational study. EBioMedicine 99:104930. doi: 10.1016/j.ebiom.2023.104930, 38168587 PMC10794922

[ref11] Puymirat E. Simon T. Cayla G. Cottin Y. Elbaz M. Coste P. . (2017). Acute myocardial infarction: changes in patient characteristics, management, and 6-month outcomes over a period of 20 years in the FAST-MI program (French registry of acute ST-elevation or non-ST-elevation myocardial infarction) 1995 to 2015. Circulation 136, 1908–1919. doi: 10.1161/CIRCULATIONAHA.117.030798, 28844989

[ref12] Sabatine M. S. Giugliano R. P. Keech A. C. Honarpour N. Wiviott S. D. Murphy S. A. . (2017). Evolocumab and clinical outcomes in patients with cardiovascular disease. N. Engl. J. Med. 376, 1713–1722. doi: 10.1056/NEJMoa1615664, 28304224

[ref13] Sanchis J. Bueno H. Minana G. Guerrero C. Marti D. Martinez-Selles M. . (2023). Effect of routine invasive vs conservative strategy in older adults with frailty and non-ST-segment elevation acute myocardial infarction: a randomized clinical trial. JAMA Intern. Med. 183, 407–415. doi: 10.1001/jamainternmed.2023.0047, 36877502 PMC9989957

[ref14] Sharma A. Zheng Y. Ezekowitz J. A. Westerhout C. M. Udell J. A. Goodman S. G. . (2022). Cluster analysis of cardiovascular phenotypes in patients with type 2 diabetes and established atherosclerotic cardiovascular disease: a potential approach to precision medicine. Diabetes Care 45, 204–212. doi: 10.2337/dc20-2806, 34716214 PMC9004312

[ref15] Van Veelen A. Elias J. Van Dongen I. M. Hoebers L. P. C. Claessen B. E. P. M. Henriques J. P. S. (2021). Percutaneous coronary intervention versus medical therapy for chronic total coronary occlusions: a systematic review and meta-analysis of randomised trials. Neth Heart J. 29, 30–41. doi: 10.1007/s12471-020-01503-0, 33064274 PMC7782674

