# Supplementary material for: Unsupervised clustering identifies distinct phenotypes in acute myocardial infarction: insights from the FAST-MI 2015 registry
Source: Front Artif Intell. 2026 Aug 19;9:1886402. doi: 10.3389/frai.2026.1886402 (PMC13536943; doi:10.3389/frai.2026.1886402)

Supplementary Table 1. Missing data in the overall FAST-MI 2015 population (n = 5,291) and variables used for clustering analysis

	Missing (%)	Included as clustering variable
**Type of establishment**	0.0%	Yes
**SOCIO-DEMOGRAPHICS**		
**Female gender**	0.0%	Yes
**Age (years)**	0.4%	Yes
**CARDIOVASCULAR RISK FACTORS**		
**BMI (kg/m²)**	4.4%	Yes
**Weight (kg)**	3.2%	No
**Arterial hypertension**	0.4%	Yes
**Dyslipidaemia**	0.7%	Yes
**Diabetes Mellitus**	0.6%	Yes
**Current smoking**	4.6%	Yes
**Comorbidity**	0.6%	Yes
**Initial presentation (first MI event)**	0.0%	Yes
**TREATMENTS IN PROGRESS AT THE TIME OF AMI**		
**Proton pump inhibitor**	0.0%	Yes
**Diuretics**	0.0%	Yes
**Betablocker**	0.0%	Yes
**Calcium channel blocker**	0.0%	Yes
**ACE inhibitor**	0.0%	Yes
**ARB**	0.0%	Yes
**Statin**	0.0%	Yes
**Levothyroxyn**	0.0%	Yes
**Anti-inflammatory drug**	0.0%	Yes
**Opioïds**	0.0%	Yes
**Antiepileptic**	0.0%	Yes
**Anxiolytics**	0.0%	Yes
**Sleeping pills**	0.0%	Yes
**Antidepressant**	0.0%	Yes
**CLINICAL PRESENTATION**		
**Diagnosis at admission (STEMI/NSTEMI)**	0.1%	Yes
**Typical chest pain**	2.5%	No
**Heart failure / Acute pulmonary edema**	3.4%	No
**Cardiac arrest**	3.4%	No
**Syncope**	3.3%	No
**Killip = III / IV**	7.8%	No
**Heart rate (bpm)**	5.2%	No
**Systolic Blood Pressure (mmHg)**	4.7%	No
**Diastolic Blood Pressure (mmHg)**	4.8%	No
**BIOLOGICAL PRESENTATION**		
**LDL-cholesterol (mmol/L)**	23.1%	No
**HDL-cholesterol (mmol/L)**	21.8%	No
**Triglycerides (mmol/L)**	54.1%	No
**C Reactive Protein (mg/L)**	23.5%	Yes *
**Leucocytes ($10^3/mm^3$)**	5.4%	No
**Red cells ($10^6/mm^3$)**	6.8%	No
**Platelets ($10^3/mm^3$)**	4.7%	No
**Haemoglobin (g/dL)**	4.0%	No
**Glycaemia (mmol/L)**	15.7%	No
**Creatinine (µmol/L)**	4.0%	Yes *
**IN-HOSPITAL MANAGEMENT**		
**Any assistance**	0.6%	No
**Coronarography performed**	0.0%	No
**PCI performed**	0.1%	No
**CABG performed**	0.1%	No
**MEDICAL TREATMENTS USED IN THE FIRST 48H**		
**Antiplatelet drugs**	0.0%	No
**Proton pump inhibitor**	0.0%	No

**Diuretics**	0.0%	No
**Betablocker**	0.0%	No
**Calcium channel blocker**	0.0%	No
**ACE inhibitor**	0.0%	No
**ARB**	0.0%	No
**ACE inhibitor or ARB**	0.0%	No
**Statin**	0.0%	No
**IN-HOSPITAL OUTCOMES**		
**Death**	0.0%	No
**Ischemic recurrence**	0.1%	No
**Atrioventricular block II/III appearance**	0.5%	No
**Mechanical complication**	0.2%	No
**Left intraventricular thrombus**	0.3%	No
**Non-compressive pericardial effusion**	0.2%	No
**Stroke / TIA**	0.2%	No
**Minor or major bleeding**	0.3%	No
**Transfusion**	0.0%	No
**Pacemaker implantation**	0.4%	No
**Defibrillator implantation**	0.5%	No
**Intensive care stay**	1.2%	No
**Length of hospital stay (days)**	0.0%	No

* included in the clustering procedure with missing values handled through a separate "unknown" category.
Percentages are calculated using the overall FAST-MI 2015 population (n = 5,291).

Supplementary Table 2 : Main characteristics compared between excluded patients and patients included for clustering analysis.

	**included in clustering analysis** *n=4,947*	**excluded** *n=344*	**p**
Age (years), *mean ± SD*	65 ± 14	70 ± 14	< 0.001
Female*, (%)*	27%	40%	< 0.001
Hypertension*, (%)*	54%	57%	0.179
Diabetes*, (%)*	22%	25%	0.196
Smoking*, (%)*	36%	33%	0.481
STEMI*, (%)*	50%	58%	0.005
Comorbidity*, (%)*	29%	27%	0.617
Killip III-IV*, (%)*	4%	8%	0.002
Revascularized*, (%)*	81%	76%	0.020
Mortality at 1 year*, (%)*	7%	15%	< 0.001

Supplementary Table 3: Sensitivity analysis of cluster profiles after exclusion of patients with missing clustering laboratory variables (CRP and creatinine)

	**Cluster 1**		**Cluster 2**		**Cluster 3**		**Cluster 4**	
	**primary analysis**	**sensitivity analysis**	**primary analysis**	**sensitivity analysis**	**primary analysis**	**sensitivity analysis**	**primary analysis**	**sensitivity analysis**
	n=1,488	*n=735*	*n=1,305*	*n=624*	*n=815*	*n=1,006*	*n=1,339*	*n=1,415*
Age (years), *mean ± SD*	68 ± 4	75 ± 3	82 ± 5	85 ± 4	44 ± 5	47 ± 6	57 ± 3	63 ± 4
Female*, (%)*	23%	30%	45%	52%	19%	18%	18%	19%
Diabetes*, (%)*	27%	29%	29%	29%	9%	12%	18%	24%
Smoking*, (%)*	24%	13%	6%	5%	73%	70%	57%	39%
STEMI*, (%)*	47%	41%	39%	37%	62%	59%	56%	50%
Comorbidity*, (%)*	32%	40%	42%	42%	17%	19%	19%	25%
Killip III-IV*, (%)*	4%	7%	8%	9%	2%	2%	2%	3%
Mortality at 1 year*, (%)*	6%	11%	16%	19%	2%	2%	2%	4%

Supplementary Figure 1 : Flow diagram of patient selection and cluster assignment

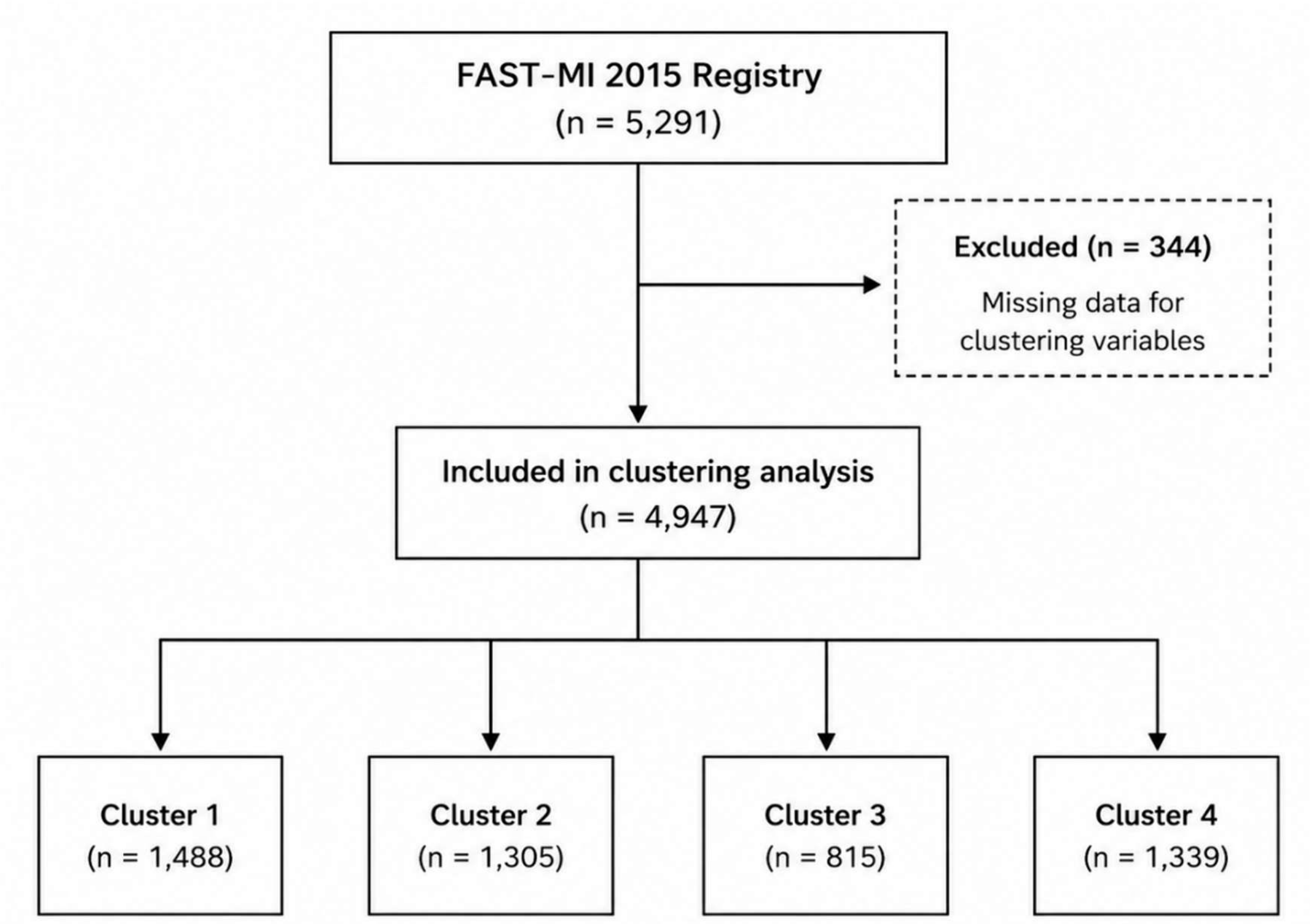

Supplementary Figure 2 : Top 15 variables contributing to cluster separation

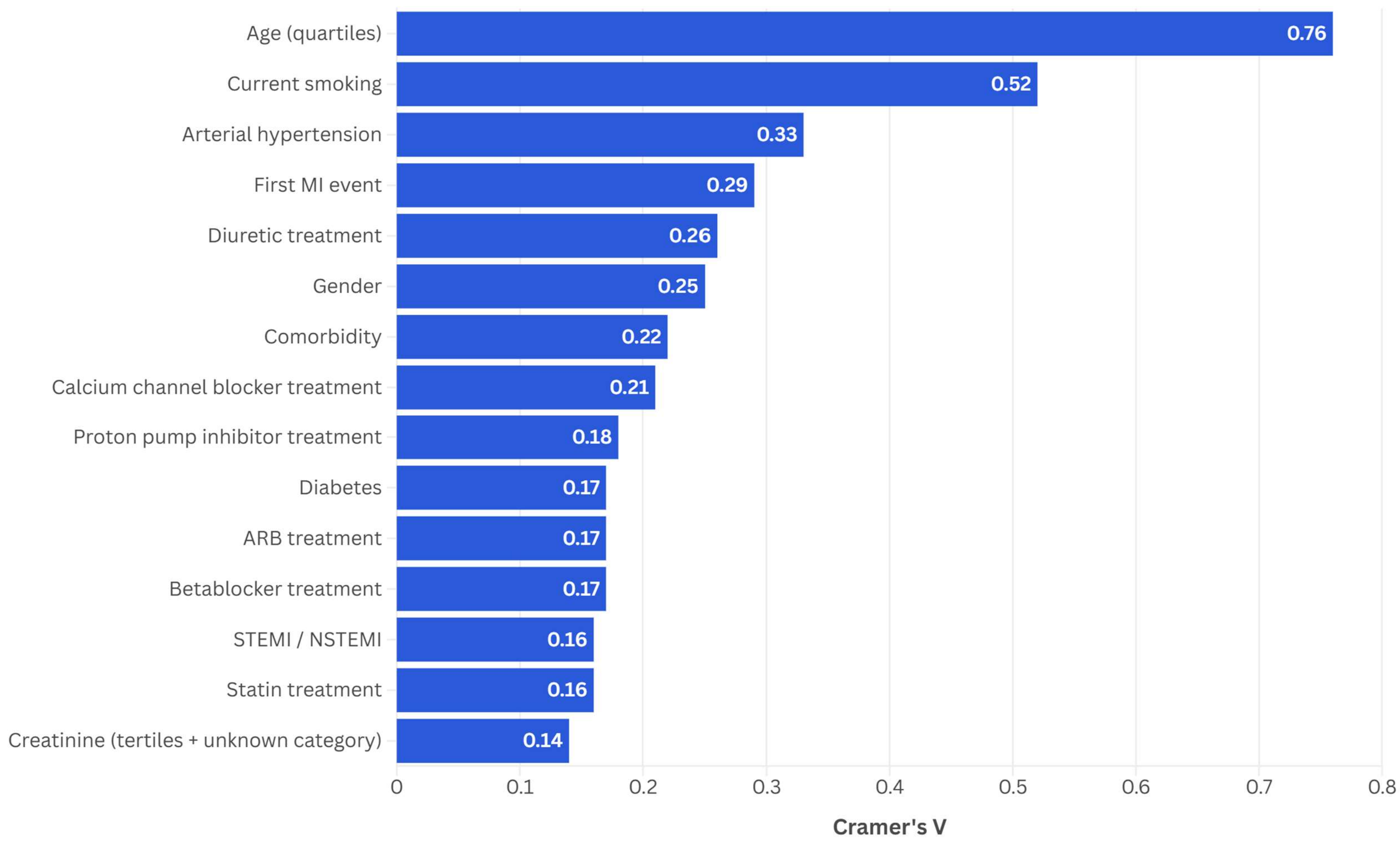

Supplement: Supplementary file 1 [file Supplementary_file_1.pdf]
